# Transcranial Doppler sonography and the effect of haematopoietic stem cell transplantation in sickle cell disease

**DOI:** 10.1186/s42466-022-00175-y

**Published:** 2022-04-04

**Authors:** Sylvia Thurn, Katharina Kleinschmidt, Irena Kovacic, Christina Wendl, Ralf A. Linker, Selim Corbacioglu, Felix Schlachetzki

**Affiliations:** 1 Institute of Diagnostic Radiology, University Medical Center Regensburg, Regensburg, Germany; 2 Department of Paediatric Haematology, Oncology and Stem Cell Transplantation, University Medical Center Regensburg, Regensburg, Germany; 3 Department of Neurology, University of Regensburg, Bezirksklinikum Regensburg, Regensburg, Germany; 4 Department of Neurology, University Medical Center Regensburg, Regensburg, Germany; 5 Center for Neuroradiology, Bezirksklinikum Regensburg and University Medical Center Regensburg, Regensburg, Germany

**Keywords:** Sickle cell disease, Stroke, Transcranial Doppler, Haematopoietic stem cell transplantation

## Abstract

**Background:**

Sickle cell disease (SCD) is one of the most prevalent monogenetic diseases worldwide and one of the most serious complications is stroke. Transcranial Doppler (TCD) demonstrated to be highly predictive for an imminent stroke by measuring blood flow velocities in the basal cerebral arteries. Currently, the only curative therapy for SCD is hematopoietic stem cell transplantation (HSCT). The aim of this study is to verify the correlation between blood flow velocities and stroke including the effect of HSCT.

**Methods:**

In our retrospective single-center study a total of 26 sickle cell patients (HbSS, HbSß^+^-thalassemia, HbSSα-thalassemia minima, HbSSα-thalassemia minor and HbSC) were analyzed between 2010 and 2016. The highest time averaged maximum mean blood flow velocity (TAMMV) measured was documented and evaluated with respect to SCD genotype and effect of HSCT. Acute and symptomatic as well as silent strokes were recorded as separate parameters.

**Results:**

In our study, ten patients had normal blood flow velocities before HSCT (six HbSS and four HbSß^+^-thalassemia patients) and 13 patients presented with abnormal TCD (eight HbSS, three HbSSα-thalassemia minima, one HbSSα-thalassemia minor and one HbSC). Thirteen of 26 study participants (ten HbSS and three HbSß^+^-thalassemia patients) received HSCT. In two patients, TAMMV in basal cerebral arteries remained “normal”, in one they remained conditional and in one TAMMV was reduced to normal. Four of 26 study participants (15.4%), including all patients with HbSS genotype, presented with a stroke, but none had “abnormal” TAMMV with TCD performed after the onset of stroke in each case. At the time we performed the TCD, the patients had already suffered the stroke.

**Conclusion:**

In our study, none of the patients with stroke displayed abnormal blood flow velocities in TCD. Yet, HSCT at this stage of the disease still had a positive effect on TAMMV. Further studies are needed whether this effect converts into reduced stroke risk at all or only selected SCD patients undergoing HSCT.

## Background

Sickle cell disease (SCD) is the most prevalent haemoglobinopathy and leading cause for stroke in children worldwide [1, 2]. An autosomal recessive mutation in the haemoglobin gene leads to the expression of sickle cell haemoglobin (HbS). The sickle cell hemoglobin arises from a point mutation in the β-globin chain of hemoglobin on chromosome 11 at position six by an amino acid substitution, resulting in the synthesis of valine (GTG) instead of glutamic acid (GAG) [3–5]. The pathophysiology of SCD results from the polymerization of HbS in the deoxygenated state [4–12].

SCD is endemic in Sub-Saharan Africa with around 200,000 births annually [3, 13]. Affected are also parts of India, Saudi Arabia, Turkey and neighboring countries in the Middle East [7, 12, 13]. Due to migration, patients affected by SCD in Germany is continuously increasing [14]. From 2007 to 2015 an increase of over 60% (from 2016 to 3216 immigrants) was observed. Approximately 3000 SCD patients are currently registered in Germany, but the real prevalence may be higher [15].

SCD is a systemic vasculopathy affecting every organ of the body. One of the most serious complications of SCD is stroke [5, 16, 17]. By the age of 20, 5–10% of those affected suffer from cerebral insult [5]. Frequently steno-occlusive lesions in the distal portion of the internal carotid artery (ICA) and the proximal portion of the middle cerebral artery (MCA) as well as microangiopathic lesions occur causing “silent” and symptomatic ischemic stroke, yet also patients suffering from hemorrhagic stroke exist [6, 18, 19].

Transcranial Doppler sonography (TCD) is the gold standard to assess blood flow velocities in the vessels of the Circle of Willis in these patients [4, 6, 16, 20]. In a landmark study, the STOP trial (stroke prevention trial in sickle cell anemia), the risk of stroke was found to be predictable measuring time-averaged maximum mean velocity (TAMMV) in the basal brain supplying arteries [3, 18]. A variety of factors in SCD seem to contribute to the development of steno-occlusive lesions in the macro- and, to a lesser degree, microcirculation: altered blood rheology leading to decreased nitric oxide production, endothelial dysfunction and inflammation, hypoxia with upregulation of several endothelial adhesion molecules, severity of hypoxia increasing blood flow and post-stenotic shear stress, and G6PD deficiency all of which leading to endothelial hyperplasia and increase of media thickness [21]. Children with blood flow velocities > 200 cm/s in the MCA and distal ICA have an increased risk of approximately 40% of developing a stroke within the following three years [16, 18, 22, 23]. Thus, TCD of the Circle of Willis is highly recommended as a routine screening tool and should be applied annually from the second to the 16th year of life [3, 7, 16, 23]. Abnormal TCD resulted in inclusion to exchange transfusions programs and reduced the incidence of stroke by up to 95% [3, 8, 17, 18].

However, the only currently available curative treatment for SCD is allogeneic haematopoietic stem cell transplantation (HSCT) [8, 24]. Patients transplanted from a HLA-identical sibling (matched sibling donor; MSD) have an overall survival between 92 and 94% and an event-free survival between 84 and 92% [24]. Since matched sibling donor or matched unrelated donor (MUD) are available in less than 20% of SCD patients, HLA-haploidentical stem cell donation is increasingly used as a realistic alternative [24].

The aim of this study is to verify the correlation between blood flow velocities and stroke including the effect of HSCT.

## Methods

This retrospective single-centre study included 26 patients with SCD who presented to the Department of Paediatric Haematology, Oncology and Stem Cell Transplantation of the Children's University Hospital Regensburg between January 2010 and September 2016. The study protocol was approved by the ethics committee of the University Hospital Regensburg (20-2015-104). Inclusion criterion was the presence of a homozygous or heterozygous SCD. Data analysis was based on electronic (SAP) and archived patient records. Patient file included all physicians’ letters, all consultative findings, all findings of the examinations carried out as well as the blood values, medications and the entire documentation of the physicians and nursing staff during an inpatient stay or the presentation in the Department.

For the study, we determined age, sex and diagnosis (genotype of SCD as well as the potential combination with an α/ß-thalassemia) of the study participants. The data were collected on a password-protected computer without internet connection in a previously created table and were subsequently analyzed.

### Transcranial Doppler sonography (TCD)

TCD examinations were performed in all patients by the same experienced board-certified stroke neurologist at the University Hospital Regensburg (FS) using a phased-array 2–3.5 MHz transducer for transcranial color-coded Duplex sonography. In order to assure comparison to standard TCD as performed by Adams et al. [19], no angle correction was used and the sample volume increased to 8 mm. The patients were examined in supine position. The anterior cerebral arteries (ACA), MCA, posterior cerebral arteries (PCA), and the distal ICA using anterior coronal scanning plane were insonated through the temporal window [25]. The vertebral arteries and the basilar artery were be assessed through the suboccipital window [6]. Subsequently, the highest time averaged maximum mean blood flow velocity (TAMMV) was documented in centimeters per second (cm/s). According to the studies by Adams et al. [18, 20] blood flow velocities < 170 cm/s were defined as “normal”, between 170 and 200 cm/s as “conditional” and > 200 cm/s as “abnormal”, the latter carrying the highest stroke risk.

TCD was performed at our University Hospital depending on the time interval from the last TCD. TCD was performed at least once in every patient without HSCT or before and/or after stem cell transplantation.

### Stroke

Information on symptomatic stroke was deduced from the documentation in the medical charts. Most patients were referred to the medical center for the haemoglobinopathy-expertise. We documented the genotype and age of the patient with any acute and past stroke and noted the region where the stroke occurred. Imaging for acute stroke diagnosis was performed at external hospitals using cerebral computer tomography (cCT) and cerebral magnetic resonance imaging (cMRI) in the follow-up phase or preceeding HSCT in order to document silent stroke events.

### Stem cell transplantation

Depending on the availability of stem cell donors, HSCT was performed as standard of care from a matched sibling donor, or in an alternative treatment regimen from a haploidentical donor. All study participants received an exchange transfusion prior to HSCT, in order to avoid sickle crisis caused by the chemotherapy during the conditioning phase [26, 27]. HSCT was performed by the Department of Paediatric Haematology, Oncology and Stem Cell Transplantation at the University Medical Center Regensburg, Germany.

## Results

### Characteristics of the study participants

We included 26 patients (ten female and 16 male) with SCD, with 16 patients suffering from homozygous SCD (HbSS) (61.5%), five with HbSß^+^-thalassemia (19.2%), three with HbSS α-thalassemia minima (11.5%), one patient with HbSS α-thalassemia minor (3.8%) and one patient with HbSC disease (3.8%). Patient age ranged from three to 34 years (median age: HbSS 12.6, HbSß^+^-thalassemia 21.2, HbSSα-thalassemia minima 4.2, HbSSα-thalassemia minor 6.4 and HbSC 4.4). One HbSS patient died post-transplant as a consequence of severe cytomegalovirus pneumonia.

### TCD: relationship with stem cell transplantation and stroke

TCD was performed in all study participants. Table 1 shows an overview of the findings related to HSCT (none or before and after HSCT) and the presence/absence of overt stroke.

**Table 1:** TAMMV without stem cell transplantation or before and after stem cell transplantation with overt stroke

Genotype	Acute stroke	TAMMV with/without HSCT	HSCT	TAMMV after HSCT
HbSS	x	Normal	x	–
HbSS	x	–	x	Normal
HbSS	x	–	x	Normal
HbSS	–	Conditional	x	Normal
HbSS	–	Conditional	x	–
HbSS	–	Normal	x	Normal
HbSß^+^-Thalassemia	–	Normal	x	–
HbSß^+^-Thalassemia	–	–	x	Normal
HbSS	–	Conditional	x	Conditional
HbSS	–	Conditional	x	–
HbSS	–	Normal	x	–
HbSS	–	Normal	x	–
HbSß^+^-Thalassemia	–	Normal	x	Normal
HbSS	x	Conditional	–	–
HbSß^+^-Thalassemia	–	Normal	–	–
HbSS	–	Normal	–	–
HbSS	–	Normal	–	–
HbSS	–	Conditional	–	–
HbSS	–	Conditional	–	–
HbSS	–	Conditional	–	–
HbSß^+^-Thalassemia	–	Normal	–	–
HbSSα-Thalassemia minima	–	Conditional	–	–
HbSSα-Thalassemia minima	–	Conditional	–	–
HbSSα-Thalassemia minima	–	Conditional	–	–
HbSSα-Thalassemia minor	–	Conditional	–	–
HbSC	–	Conditional	–	–

Normal TAMMV were defined as blood flow velocities < 170 cm/s. Conditional TAMMV were blood flow velocities between 170 and 200 cm/s

### Transcranial Doppler sonography and stem cell transplantation

Of the 13 patients who received HSCT (ten HbSS and three HbSß^+^-thalassemia patients) TCD was performed in ten patients before HSCT. Among them, six patients (four patients with the HbSS genotype and two patients with HbSß^+^-thalassemia) showed “normal” TAMMV before HSCT. Four patients (all of the HbSS genotype) showed “conditional” TAMMV. No “abnormal” TAMMV with highest stroke risk was found.

After stem cell transplantation, TCD was performed (four months post-HSCT) in seven patients. The remaining transplanted patients received their follow-up checks in hospitals close to their home. Among the seven patients presented to us six patients showed “normal” TAMMV (four patients of HbSS genotype and two patients with HbSß^+^-thalassemia) and one HbSS patient showed “conditional” TAMMV.

Four patients received TCD before and after HSCT. In one patient, the values decreased from conditional to normal TAMMV after HSCT. In two patients, “normal” TAMMV were detected before and after HSCT and in one patient, “conditional” values occurred both, before and after transplantation (see Table 1).

Among 13 patients who did not receive HSCT, “normal” TAMMV were measured in four patients (two HbSS and two HbSß^+^-thalassemia patients) and “conditional” TAMMV could be derived in nine patients (four HbSS, three HbSSα-thalassemia minima patients, one patient with HbSSα-thalassemia minor and one with HbSC).

Overall, study participants were less likely to have “normal” TAMMV before transplantation (six out of ten, 60.0%) and without transplantation (four out of 13, 30.8%) than after HSCT (six out of seven, 85.7%).

### Stroke

**Fig. 1:**
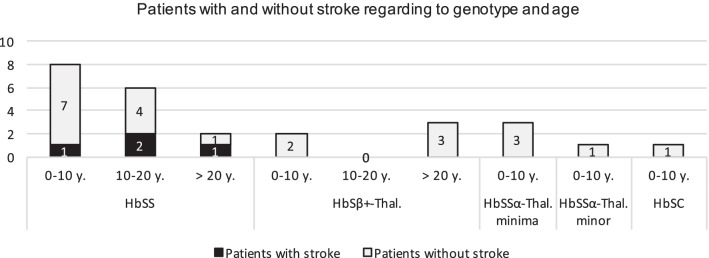
Patients with and without overt stroke regarding genotype and age (at the end of data collection or, in patients with stem cell transplantation, age at transplantation). The number of patients with a certain genotype of SCD and within a certain age range is indicated in the columns. Patients with overt stroke are black-marked, patients without stroke are grey-marked

Four of 16 HbSS patients (25.0%) and 15.4% of the study population were diagnosed with stroke. Among them, one infarct in the MCA territory, one infarct in both the ACA and MCA territory, one patient with sinus thrombosis in combination with MCA territory infarction and one patient with non-aneurysmatic subarachnoid hemorrhage with bilateral infarcts (frontal bilaterally, occipital bilaterally, in the central and precentral region bilaterally, and to a lesser extent cerebellar bilaterally), probably due to vasospasm, was found. There was a higher prevalence for stroke with increasing age, see Fig. 1.

Three of four patients received HSCT. In this group, no recurrent stroke occurred until the end of data collection.

### Relationship between TAMMV and stroke

In one patient, “normal” TAMMV could be determined before HSCT, but no TCD was performed after HSCT. Two patients with symptomatic stroke did not receive TCD before HSCT but showed “normal” values after HSCT. The patient with symptomatic stroke, who did not receive HSCT, showed “conditional” TAMMV.

### Relationship between TAMMV and stem cell transplantation

Four patients underwent TCD before and after HSCT, rendering a comparative analysis feasible. Table 2 illustrates the highest measured TAMMV and the changes of blood flow velocities for these four patients with regard to genotype.

**Table 2:** Changes in TAMMV before and after HSCT regarding to genotype

Genotype (age at HSCT)	TAMMV before HSCT (median)	TAMMV after HSCT (median)	Changes
HbSS (age 4 years)	Conditional	Conditional	→
HbSS (age 18 years)	Normal	Normal	→
HbSS (age 22 years)	Conditional	Normal	↘
HbSß^+^-Thal. (age 27 years)	Normal	Normal	→

Three patients did not show any changes in blood flow velocities after HSCT. TAMMV remained “conditional” in a 4-year-old HbSS patient and TAMMV remained “normal” in an 18-year-old HbSS patient and in a 27-year-old patient with HbSß^+^-thalassemia. In a 22-year-old HbSS patient, TAMMV decreased from “conditional” to “normal”.

**Fig. 2:**
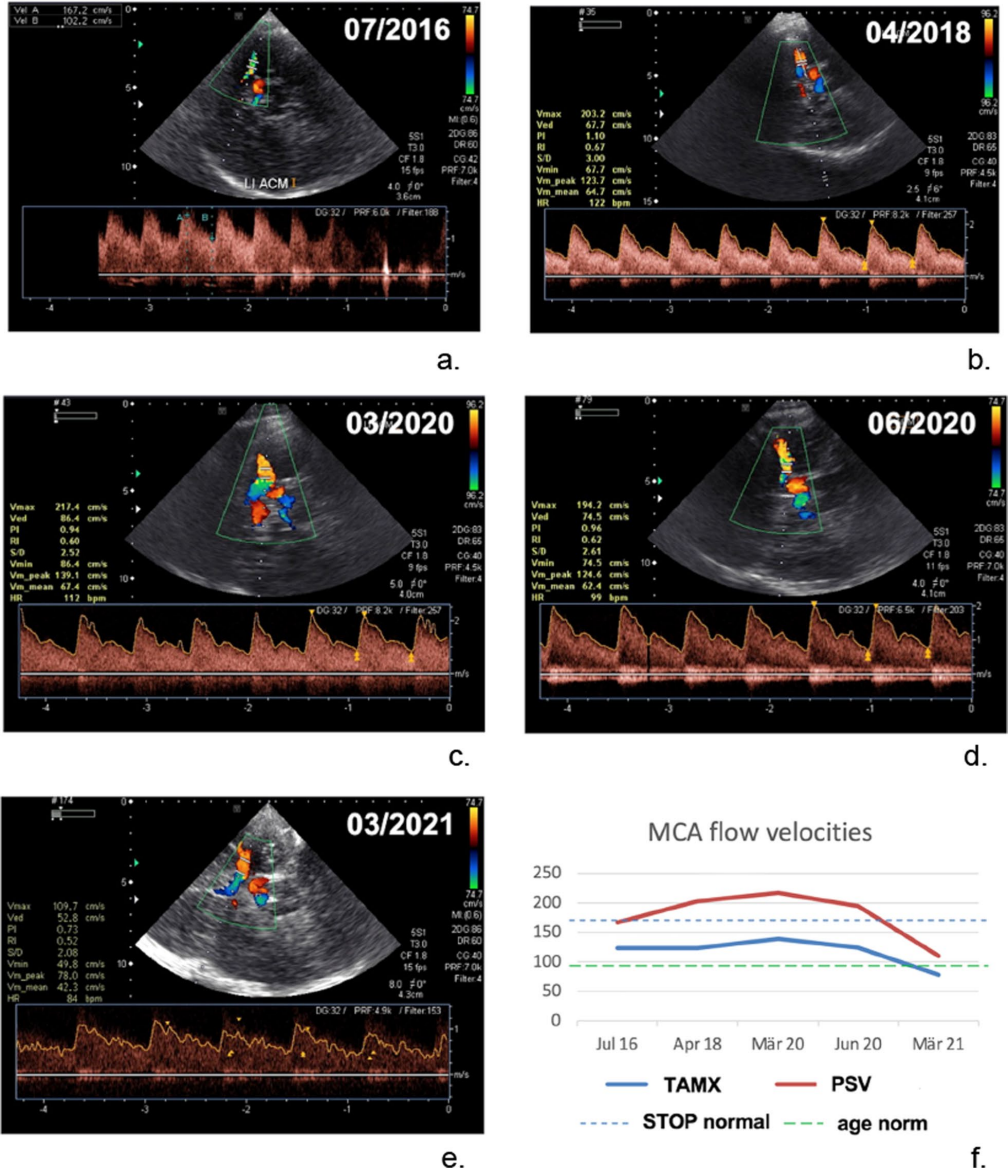
Transcranial Doppler sonography of a 14-year-old SCD-patient without stem cell transplantation. Measurements were taken in the middle cerebral artery (MCA). There was a long-term follow-up from 07/2016 to 03/2021 (see **a**–**e**). A total of 5 TCDs were performed. **f** Shows the time course of PSV (peak systolic velocity) and TAMX (time averaged maximum velocity, TAMMV). As abnormal blood flow velocities with a TAMX of 217 cm/s were derived in the TCD in 03/2020, we performed a control in short-term interval in 06/2020. Here, a TAMX/TAMMV of < 200 cm/s ("conditional") was shown again

The following Fig. 2 shows the TCD and also the time course of PSV (peak systolic velocity) and TAMX (time averaged maximum velocity, TAMMV) over almost 5 years of a 14-year-old SCD-patient.

## Discussion

### Stroke

In our retrospective study, TCD in SCD patients after stroke revealed only “normal” to “conditional” cerebral blood flow velocities, probably due to the small sample size and the strict transplant indications due to other SCD related complications. After HSCT, a slight improvement in measured blood flow velocities in TCD was observed adding confidence on this therapy as the optimal long-term treatment strategy for these patients.

Patients with SCD have an increased risk of developing stroke [17]. In our study, only patients with HbSS genotype were affected by symptomatic stroke. According to a clinical trial of the Cooperative Study of Sickle Cell Disease [28] with a total of 4.082 sickle cell patients, that was conducted at 23 hospitals from October 1978 to September 1988, the HbSS genotype showed the highest prevalence of stroke among the diverse genotypes of SCD at 4.01% (mean age: 14.1 years), followed by HbSß^0^-thalassemia (2.43%, mean age 15.6 years), HbSß^+^-thalassemia (1.29%, mean age 16.2 years) and HbSC (0.84%, mean age 13.5 years). For HbSSα-thalassemia patients, we are not aware of a literature value for prevalence, but Ohene-Frempong et al. describes a protective effect of an additional α-thalassemia in patients with homozygous SCD [28].

Thus, in a similar age group, our study shows a six-fold higher prevalence among HbSS patients that may be explained by a "selection bias" in our study group. No acute and symptomatic stroke was observed in the other genotypes, a finding consistent with the literature given the small study population.

### Transcranial Doppler sonography: relationship with stem cell transplantation and stroke

#### Relationship between TAMMV and stroke

Despite a high prevalence of stroke among HbSS patients, TAMMV did not show “abnormal” values in any of our study participants, indicative of an annual stroke risk increase from 0.5–1% (“conditional”) to 10% [16, 20]. However, TCD was performed only after the occurrence of an overt stroke in each case with three patients showing “normal” and one patient displaying “conditional” TAMMV (see Table 1). In fact, most of the patients with stroke presented with “normal” blood flow velocities, a characteristic attributed normalization within the time interval from stroke to presentation in our clinic.

#### Relationship between TAMMV and stem cell transplantation

The current study reveals a “normalization” of TAMMV from 60.0% before HSCT to 85.7% after HSCT. This increased TAMMV values in SCD are linked to low Hb, young age and low arterial desaturation [22]. After HSCT, depending on the donor approximately 30% HbS is present (before HSCT, depending on the genotype, up to 90%) [16, 29]. Thus, and next to age, higher Hb and normal arterial desaturation after HSCT contribute to normalization of cerebral blood flow. However, it must be noted that the effect of HSCT on TAMMV might be higher when more high risk SCD patients, especially younger patients are investigated and transplanted, and therefore the value of TCD surveillance might be higher. In addition, further studies should extend the time interval after HSCT to at least 6 months to exclude long term complications. However, in our institution this has been become standard practice given good patient compliance and availability. However, our data are in line with a study by Bernaudin et al. who performed a non-randomized controlled trial including 67 children with SCD and HCST including 7 patients with stroke [30]. After one year intracranial TAMMV in the matched sample were significantly lower on average in the transplantation group (129.6 cm/s) vs the standard care group (170.4 cm/s; difference, − 40.8 cm/s [95% CI, − 62.9 to − 18.6]; P < 0.001) which is in line with our data. Large registries on SCD patients should implement radiological and TCD databanks to proper phenotype neurovascular sequelae after HCST to determine its value on long term outcome. In the future, high field MRI with angiography in conjunction with TCD may be the ideal combination to better characterize cerebrovascular pathology and brain parenchymal changes in SCD and serve as another surrogate marker for therapeutic strategies [31].

## Conclusion

This work aimed to investigate the relationship between altered blood flow velocities in the vessels of the Circle of Willis and stroke in SCD patients. Measurement of the blood flow velocities by TCD is still considered the gold standard for determining risk of stroke in SCD patients [6, 8, 16, 18, 32]. The patient collective in our clinic presented primarily with the aim for HSCT and not with acute stroke. Thus, we can only assume that intracranial blood flow velocities were higher before the onset of stroke and normalized after stroke [18, 33]. It should be noted that, in contrast to the USA and neighboring European countries, where TCD is now part of routine screening in children with SCD [22], this examination is unfortunately not standard in all patients with SCD in Germany [7, 34]. Heightened awareness of SCD and stroke risk may lead to earlier curative treatment such as HSCT including serial TCD examinations, and thus reduced stroke risk as HSCT is considered to be the only established curative treatment for SCD [4, 8, 16, 23].

Finally, it remains to be mentioned that our analysis is limited by the small patient cohort and by the retrospective character of the study. A prospective study with a larger study population is indispensable for validation if normalization of cerebral blood flow velocities may actually serve as a surrogate for decreased stroke risk after HSCT.

## Data Availability

The datasets generated during this study are available from the corresponding author upon reasonable request.

## Abbreviations

HSCT: Haematopoietic stem cell transplantation
SCD: Sickle cell disease
TAMMV: Time averaged maximum mean blood flow velocity
TAMX: Time averaged maximum velocity
TCD: Transcranial color-coded Duplex sonography
HbS: Sickle cell haemoglobin
ICA: Internal carotid artery
MCA: Middle cerebral artery
ACA: Anterior cerebral artery
PCA: Posterior cerebral artery
cCT: Cerebral computed tomography
cMRI: Cerebral magnetic resonance imaging
MSD: Matched sibling donor
MUD: Matched unrelated donor
